# Characteristics of older adult hospitalized patients with bronchial asthma: a retrospective study

**DOI:** 10.1186/s13223-021-00628-0

**Published:** 2021-12-03

**Authors:** Yuan Zhang, Lewei Huang

**Affiliations:** 1 Jinzhou Medical University, Jinzhou, China; 2 Department of Respiratory Diseases, General Hospital of Northern Theater Command, 83 Wenhua Road, Shenhe District, Shenyang, China

**Keywords:** Bronchial asthma, Hospitalization, Older adult patients

## Abstract

**Background:**

Bronchial asthma is a chronic inflammation of the airways. Older adult patients with bronchial asthma are defined as patients older than 65 and with a previous or current clear diagnosis of asthma. The purpose of this study was to determine the characteristics of older adult hospitalized patients with bronchial asthma.

**Methods:**

We retrospectively analyzed the data from patients with bronchial asthma admitted to the General Hospital of the Northern Theater Command from September 2018 to January 2020. We divided them into the older adult (≥ 65 years) and the younger (< 65 years) groups. We compared the clinical and epidemiological characteristics of the two groups.

**Results:**

There were 181 inpatients with bronchial asthma, including 41 older adult patients, accounting for 22.7%. There were significant differences in age, sex, smoking, duration of disease, age at diagnosis of asthma, hospital stays, hospitalization costs, number of acute attacks 1 year before admission, number of hospitalizations in our hospital one year before admission, asthma control test score, forced expiratory volume in 1 s (FEV1), FEV1/FVC, the severity of acute attacks, comorbidities, and inhaled corticosteroid dose between the two groups. There were many older adult patients with asthma (mostly late-onset asthma). The hospitalization costs were high. Most patients had many comorbidities, poor asthma control, severe attack, and heavy economic burdens.

**Conclusion:**

Attention should be focused on achieving asthma control in older adult patients to improve their quality of life and reduce their economic burdens.

## Background

Bronchial asthma is characterized by chronic airway inflammation and hyperresponsiveness. Chronic airway inflammation results from interactions among various inflammatory cells (including eosinophils, mast cells, Th1, Th2, and regulatory T cells), cytokines (including interleukin [IL]-4, IL-5, IL-9, and IL-13), and inflammatory mediators (including thromboxane 2 and leukotriene B4) [1]. Hyperresponsiveness of the airway implies that the trachea is highly sensitive and engages in excessive or premature contraction responses to various stimuli. Airway hyperresponsiveness, bronchoconstriction, and mucus secretion ultimately lead to airway remodeling. There are approximately 315 million asthma patients worldwide, and the prevalence is 0.7–11.9% [2].

By contrast, the prevalence of asthma in older adult patients is 6–17% and is increasing, especially in older women. Severe asthma accounts for 13.9%, and its treatment cost is high, severely increasing social burdens [3, 4]. Asthma mortality rates are high among older adults, often related to insufficient diagnosis and delayed treatment [5]. The complexity of asthma, the baseline decline of lung function in the older adults (which is heterogeneous among individuals and varies greatly with age), and differential responses to treatment also complicate diagnosis and treatment in this population. Various asthma phenotypes may make treatment more difficult. The disease is more severe, less sensitive to glucocorticoids, and more prone to airway remodeling. Different phenotypes require different treatments, and most older adult people have late-onset asthma (usually after 40 years of age or older) [6]. Therefore, the proper treatment requires an appropriate diagnosis.

Asthma control in older adults depends on many factors. A previous prospective cohort study analyzed the relationship between asthma control and compliance in older adults and young adults with asthma and found that comorbidities had a negative impact on treatment compliance [7]. Asthma in the older adult differs from asthma in the young in many other respects, including genetic susceptibility, environmental influences, pathogenesis, type of airway inflammation, course of the disease, comorbidities, hospitalization rate, and treatment outcomes [7, 8].

Asthma progression leads to diminished quality of life, characterized by limited activities and loss of the ability to function independently and socially. Repeated hospitalization is associated with the deteriorated condition, making treatment more difficult. This phenomenon worsens the outcomes and aggravates the economic burden on patients and their families. Most studies focused on children and adults or targeted patients without comorbidities. Therefore, in the present study, we analyzed the prevalence and characteristics of older adult patients with bronchial asthma to help achieve better outcomes for these patients.

## Methods

### Patients

The medical records of inpatients newly diagnosed or previously diagnosed with bronchial asthma at our hospital from September 1, 2018, to January 31, 2020, were retrospectively analyzed. The inclusion criteria were (1) diagnosed with bronchial asthma according to the Guidelines for Prevention and Treatment of Asthma in China, and (2) between 18 and 85 years old. Patients admitted with a diagnosis of asthma but not an acute exacerbation of asthma were excluded. Patients were divided into two groups, younger (age < 65 years) and older adult (age ≥ 65 years). This study was approved by the medical ethics committee of North Hospital. As it was a retrospective study, the requirement for informed consent was waived.

### Data collection

General data were collected, including (1) number of admissions, name, sex, age, home address, insurance type, hospitalization cost, and length of stay, (2) number of acute attacks over 1 year before admission, the number of emergency visits in our hospital over 1 year before admission, the number of hospitalizations in our hospital over 1 year before admission because of acute attacks, the regular use of inhaled corticosteroids (ICS), asthma control test (ACT) score [9], course of disease, age at diagnosis of asthma, history of allergy, smoking history, body mass index, the severity of the attacks, and comorbidities, and (3) eosinophil count, eosinophil percentage, serum total immunoglobulin E (IgE), forced expiratory volume in one second (FEV1), and FEV1/forced vital capacity (FVC). Pulmonary ventilation dysfunction was defined as a variety of respiratory system conditions and related tissue lesions resulting in decreased pulmonary ventilation function phenomenon, divided into restrictive ventilation dysfunction, obstructive ventilation dysfunction, and mixed ventilation dysfunction.

### Statistical analysis

SPSS 19.0 was used for statistical analysis. Quantitative data were expressed as mean ± standard deviation and compared using the t-test or Mann–Whitney U-test. Qualitative data were expressed as frequency or percentage and were compared using the chi-square test. P < 0.05 was considered statistically significant.

## Results

### Comparison of general information

We analyzed the data of 181 hospitalized patients with bronchial asthma. There were 41 patients in the older adult group (14 males and 27 females) and 140 in the younger group (74 males and 66 females). The older adult group had a significantly lower BMI than the younger group, but significantly longer smoking history, older age at asthma onset, and longer years of disease course; care for the older adult group was significantly more expensive than in the younger group (all P < 0.05) (Table 1).

**Table 1:** Demographic data

	Older adult group n = 41	Younger group n = 140	P
Age (years)	69.9 ± 4.8	46.1 ± 14.9	< 0.001
Sex, n (%)			0.035
Male	14 (34.2)	74 (52.9)	
Female	27 (65.9)	66 (47.1)	
BMI (kg/m^2^)	24.2 ± 3.5	24.9 ± 3.7	0.615
Smoking (packs/year)	32.5 ± 18.4	15.5 ± 13.9	0.003
Age at asthma diagnosis (years)	57.4 ± 13.7	38.9 ± 14.6	< 0.001
Disease duration (years)	12.6 ± 12.9	7.2 ± 9.5	0.004
Type of asthma, n (%)			< 0.001
Early-onset asthma	3 (7.3)	67 (47.9)	
Late-onset asthma	38 (92.7)	73 (52.1)	
History of allergy, n (%)			0.759
Yes	13 (31.7)	48 (34.3)	
No	28 (68.3)	92 (65.7)	
Current home address, n (%)			0.258
City	38 (92.7)	137 (97.9)	
Rural area	3 (7.3)	3 (2.1)	
Insurance, n (%)			> 0.99
Yes	39 (95.1)	132 (94.3)	
No	2 (4.9)	8 (5.7)	
Hospital stays (days)	11.1 ± 2.2	10.1 ± 3.4	0.022
Hospitalization costs (yuan)	9465 ± 2624	7663 ± 3332	< 0.001

### Comparison of onset and ACT scores between the two groups

Older adult asthmatics had significantly more acute attacks and emergency room visits, and the number of admissions to our hospital differed between groups. The ACT scores were significantly worse in the older adult group than in the younger group (Table 2).

**Table 2:** Clinical data over 1 year before admission

	Older adult group n = 41	Younger group n = 140	P
Number of acute attacks			< 0.001
≤ 1	6 (14.6)	79 (56.4)	
2	24 (58.5)	44 (31.4)	
≥ 3	11 (26.8)	17 (12.1)	
Emergency visits to our hospital			0.192
Yes	22 (53.7)	59 (42.1)	
No	19 (46.3)	81 (57.9)	
Number of hospitalizations in our hospital			0.031
0	28 (68.3)	104 (74.3)	
1	13 (31.7)	24 (17.1)	
≥ 2	0	12 (8.6)	
Regular use of ICS			0.38
Yes	15 (36.6)	62 (44.3)	
No	26 (63.4)	78 (55.7)	
ACT score			0.001
< 15	14 (34.2)	27 (19.3)	
15–20	24 (58.5)	59 (42.1)	
> 20	3 (7.3)	54 (38.6)	

All data are shown as n (%)

### Comparison of laboratory test results

There were no differences in IgE, EO count, and EO% between the two groups (all P > 0.05) (Table 3). FEV1 and FEV1/FVC were lower in the older adult group than in the younger group (P = 0.002 and P = 0.003) (Table 3).

**Table 3:** Eosinophil and pulmonary function data

	Older adult group n = 41	Younger group n = 140	P
IgE (IU/mL, logarithm)	2.27 ± 0.766	2.20 ± 0.498	0.597
EO (10^9^/L)	0.10 ± 0.144	0.16 ± 0.316	0.725
FEV1 (L)	1.38 ± 0.584	2.15 ± 0.769	0.002
EO (%)	1.76 ± 3.860	2.79 ± 4.700	0.477
FEV1/FVC (%)	61.75 ± 9.021	71.19 ± 8.217	0.003

*EO* eosinophil, *FEV1* forced expiratory volume in 1 s, *FVC* forced vital capacity

### Comparison of disease severity

The older adult had significantly more severe bronchial asthma attacks than the younger group (P = 0.015) and had significantly more comorbidities (P < 0.001) (Table 4). There were no differences in disease severity or the degree of pulmonary ventilation dysfunction (P = 0.437). In the older adult group, 12 (29.3%) used low-dose ICS, 24 (58.5%) used moderate-dose ICS, and five (12.2%) used high-dose ICS. In the younger group, 88 (62.9%) used low-dose ICS, 50 (35.7%) used moderate-dose ICS, and two (1.4%) used high-dose ICS (P < 0.001) (Table 4).

**Table 4:** Disease description data

	Older adult group n = 41	Younger group n = 140	P
Severity of acute attacks			0.015
Mild	3 (7.3)	26 (18.6)	
Moderate	18 (43.9)	80 (57.1)	
Severe	16 (39.0)	25 (17.9)	
Extremely severe	0	1 (0.7)	
Number of underlying diseases			< 0.001
< 3	12 (29.3)	89 (63.6)	
3–5	24 (58.5)	47 (33.6)	
More than 5	5 (12.2)	4 (2.9)	
Comorbidities			0.015
Allergic rhinitis	6 (14.6)	31 (22.1)	
Hypertension	19 (46.3)	24 (17.1)	
Diabetes	9 (22.0)	7 (5.0)	
Coronary heart disease	8 (19.5)	16 (11.4)	
Pulmonary ventilation dysfunction			0.437
Mild	2	33	
Moderate	2	17	
Moderate-severe	4	12	
Severe	4	22	
Critical severe	3	14	
ICS dose			0
Low dose	12 (29.3)	88 (62.9)	
Moderate dose	24 (58.5)	50 (35.7)	
High dose	5 (12.2)	2 (1.4)	

All data are shown as n (%)

## Discussion

In the present study, we found that the number of older adult patients with late-onset asthma was significantly higher than in younger patients. Bronchial asthma can be divided into early-onset (< 40 years old) and late-onset (≥ 40 years old). Older adults had more late-onset bronchial asthma than younger adults. The age of onset of asthma has important implications for outcome [10]. The older adults often had repeated attacks, suggesting a gradually worsening trend. Pulmonary function decreases with age, and this natural decrease might affect the outcomes of asthma.

Indeed, a 10-year study enrolling 4,248,813 patients showed that the incidence of asthma in women was higher than in men and that the incidence of asthma increased with age [11]. The reason might be related to low education level and low income [12]. Unfortunately, data about education were not available for this study. Furthermore, it has become more socially acceptable for women to smoke [13, 14]. In addition, the life expectancy of men is shorter than women due to a higher incidence of cardiovascular diseases [15, 16]. Other studies showed that sex differences were closely related to smoking and pulmonary dysfunction, consistent with the result that middle-aged and older adult female patients were more frequent than older adult male patients in this study [17]. These findings suggest that substantial attention should be paid to older adult female patients.

In the present study, 36.6% of the older adults regularly used ICS, and 7.3% of the older adults had good bronchial asthma control. Medication compliance and good inhalation techniques are important for the long-term control of older adult asthmatics. A study showed that poor compliance and improper use of inhalation devices often occurred in the older adults with low educational levels, which may also cause poor asthma control in the older adults [18]. Of course, cognitive impairment and dementia can decrease compliance, and the prevalence of cognitive impairment increases with age in Chinese [19, 20], as in other populations. Access to inhalation devices can also affect compliance. In this study, most patients had insurance to cover medication, but in other parts of the world where there is no universal coverage for medications, this can reduce compliance. Another study showed that the severity of asthma attacks was related to the number of asthma triggers, and asthma patients with more triggers were more likely to have severe acute asthma attacks [21]. Therefore, in order to slow the deterioration of older adult patients with bronchial asthma, it is essential to help them achieve self-management, timely detection, and effective avoidance of exposure to allergens. Older adults need help establishing good daily behavior habits and forming adequate disease awareness [22]. Six steps for asthma management might successfully reduce the deterioration of their disease [23].

A study showed that over a 1-year period, the rate of emergency visits in the older adults was higher than the hospitalization rate for asthma (10.6% vs. 5.7%) [24]. The present study also showed that the emergency visit rate was higher than the hospitalization rate (53.7% vs. 31.7%), and some patients were more willing to choose emergency department visits to reduce the frequent occurrence of daytime asthma symptoms [25]. Therefore, assessing bronchial asthma control in older adult patients should consider the emergency department visit rate combined with the hospitalization rate. Several considerations must be taken into account regarding emergency department visits and hospitalization between younger and older patients. Younger patients usually work, and going to the hospital often means missing work. Many older adults live alone and are more prone to visit the hospital out of fear. Comorbidities might prevent older adults from walking to the hospital, and many of them might be unable to afford a taxi or an ambulance, even less owning a car.

The use of high-dose ICS can give the impression that poorly controlled patients are well controlled. Long-term exposure to air pollution increases the hospitalization rate of older adult asthmatics [26]. Air pollution is high in cities in China and affects the health of older adults [27], and most patients in this study were living in urban areas, without differences between the younger and older adult groups. Of note, contrary to Western countries, many Chinese homes still rely on coal for heating and cooking, contributing to air pollution [28]. In addition, the recent shift in economic policies in China led to a sharp increase in the number of cars, also contributing to air pollution [29]. Living conditions (hygiene and poverty) and living environment (pets, dirt, and hoarding) can also affect asthma, but no data were available about them in this study. These and other findings suggest that, when evaluating the control of older adult patients with asthma, clinicians should consider several factors, including the number of acute attacks, the rate of emergency visits, hospitalizations, the dose of ICS, and the quality of the living environment. A comprehensive geriatric assessment might be indicated to deal with environmental factors, social isolation, and cognition.

Older adults with bronchial asthma had more comorbidities; hypertension and diabetes were common, and allergic diseases were relatively rare [30]. The relatively rare occurrence of allergic rhinitis in older adult asthmatic patients might be related to the decline in the body function of the older adults and their insensitivity to allergic reactions. Johanna et al. [31] reported that most asthma patients after the age of 40 were non-allergic. In the present study, 46.3% of older adults had hypertension, 22.0% had diabetes, and the proportion of allergic rhinitis in older adults was lower than in the younger group (14.6% vs. 22.1%). These comorbidities might affect the clinical manifestations of older adult patients with bronchial asthma. The interaction between diseases might lead to atypical clinical manifestations in older adult patients with bronchial asthma. It was a characteristic of older adult patients with asthma to have more comorbidities. The interaction between diseases might be why the rate of emergency visits and hospitalization of older adult patients with bronchial asthma was higher than in younger patients.

We found that the peripheral blood eosinophil counts, FEV1, and FEV1/FVC of older adult patients with bronchial asthma were lower than those of the younger patients, except serum total IgE levels. This finding is consistent with the study of Bastsetseg, who showed the role of eosinophil-induced airway immune inflammation in asthma [32]. In the present study, older adult asthmatic patients smoked 32.5 packs/year, and 26.7% (4/15) had severe pulmonary dysfunction. It is highly likely that smoking is related to changes in lung structure and function in older adult asthmatic patients, including decreased chest wall compliance, declined alveolar elastic retraction, and reduction of respiratory muscle length, all of which result in the decline of lung function [33]. Declining lung function in older adult patients with asthma, coupled with the damaging effect of smoking on lung function [34], leads to the deterioration of the condition of older adult patients with bronchial asthma.

## Conclusion

The frequency of bronchial asthma among older adult patients is high, and more so in women than men. Older adult patients with asthma have a long course of illness and tend to be heavy smokers. They are diagnosed late and have poor asthma control. Older adult patients with asthma have many comorbidities, poor lung function, and severe outcomes suffering attacks. Few of the older adults regularly use ICS. Clinicians should pay more attention to older adult patients with asthma, encourage them to quit smoking, educate older patients about the need for controlling asthma, assess cognition, assess the understanding of using the devices to assure compliance, educate about the importance of rest, avoiding respiratory infections, and exercising, and perform comprehensive geriatric assessments. Doing so might lead to better management of older adult patients with bronchial asthma.

## Data Availability

All data generated or analyzed during this study are included in this published article.

## Abbreviations

ICS: Inhaled corticosteroids
ACT: Asthma control test
IgE: Immunoglobulin E
FVC: Forced vital capacity
